# Intravaginal estrogen management in postmenopausal patients with vaginal squamous intraepithelial lesions along with CO_2_ laser ablation: A retrospective study

**DOI:** 10.1515/biol-2022-0621

**Published:** 2023-08-08

**Authors:** Shengyao Lei, Congquan Wu, Siyi Zhong, Yanmei Liu, Ke Peng, Xiao Han, Jialing Chen, Chunlan Li, Shujun Gao

**Affiliations:** Department of Gynecology, Center of Diagnosis and Treatment for Cervical & Uterine Cavity Diseases, Obstetrics and Gynecology Hospital of Fudan University, No. 419 Fangxie Road, Huangpu District, Shanghai 200011, China; Shanghai Key Laboratory of Female Reproductive Endocrine-Related Disease, Fudan University, Shanghai 200011, China; Department of Gynecology, Children’s Hospital of Anhui Province, No. 39 East Wangjiang Road, Hefei 230022, China

**Keywords:** vaginal squamous intraepithelial lesion, estrogen, post-menopause

## Abstract

This study aims to investigate the influence of topical estrogen management in postmenopausal patients who had undergone CO_2_ laser ablation for vaginal squamous intraepithelial lesions (SILs). The clinical data of 211 postmenopausal women with vaginal SILs were reviewed. Patients were divided into two groups by 2-month different management: Group 1 (intervention group): patients were treated with estrogen cream 0.5 g every other day and Group 2 (control group): no topical agent was used for the treatment of patients. In low-grade squamous intraepithelial lesions (LSILs), the response rates for patients in the intervention group and the control group were 49.1% (27/55) and 54.2% (16/48), respectively; human papillomavirus (HPV) status turned negative in 12 (12/38, 31.6%) patients of the intervention group and in 15 (15/35, 42.9%) patients of the control group. In high-grade squamous intraepithelial lesions (HSILs), the response rates for patients in the intervention group and the control group were 72.4% (42/58) and 78.0% (39/50), respectively, nearly 1.5 times higher than those of the LSIL patients; 22 (22/54, 40.7%) patients of the intervention groups and 12 (12/46, 26.1%) patients of the control group cleared the HPV infection. In postmenopausal patients, local use of estrogen cream improves the recognition of lesions and is conducive to precision medicine.

## 1 Introduction

A vaginal squamous intraepithelial lesion (SIL) is a relatively uncommon disease, with an annual incidence rate of 0.2–2/100,000 [1]. No consensus has been reached on the initial treatment and post-treatment management. CO_2_ laser ablation has been widely used over the years as a primary treatment due to its safety, minimal invasiveness, repeatability [2], and effectiveness [3,4]. However, studies revealed that vaginal SIL had a high recurrence rate (3.5–13.8%), persistence rate (6.4–13.8%), and malignant progression rate (2–14.3%) in the menopausal period [1,5,6]. Vaginal SIL tends to develop during women’s menopausal period [7,8]. Long-term follow-up results supported that menopausal status was a risk factor for vaginal SIL progression (odds ratio = 10.8; 95% CI = 1.3–87.5) [9]. The evidence above suggests that lower estrogen levels could be a potential risk factor for postmenopausal women affecting CO_2_ laser ablation efficacy [10,11]. Usually, estrogen supplement is used to alleviate postmenopausal-associated symptoms [12], but as adjuvant therapy for vaginal SIL, its efficacy must be further evaluated. So far, one retrospective study assessed the outcomes of patients treated for only vaginal high-grade squamous intraepithelial lesions (HSILs), with intravaginal estrogen alone and other treatment modalities (including ablation) with estrogen or not. Although their findings suggested that intravaginal estrogen was potential, no detail was provided about the proportion and effect of each modality [13]. Considering the confounding factors in their outcomes, the impact of topical estrogen on vaginal SIL after CO_2_ laser ablation is still unclear.

Persistent high-risk (H-R) HPV infection plays an important role in precancerous and cancerous lesions of the vagina [14]. Currently, attention has been devoted to addressing that HPV infection and cervical lesions prefer a vaginal dysbiosis characterized by depleted lactobacilli [15]. Estrogen can positively influence the dominance of lactobacilli to help HPV clearance [10]. To date, few studies have taken care of whether vaginal estrogen medication could help the prognosis of vaginal SIL and HPV clearance among postmenopausal women.

To better understand the effect of estrogen administration on HPV clearance and treatment efficacy, we retrospectively analyzed 211 postmenopausal vaginal SIL patients who received CO_2_ laser surgery (combined with or without estrogen administration).

## 2 Materials and methods

### 2.1 Patients

**Figure 1:**
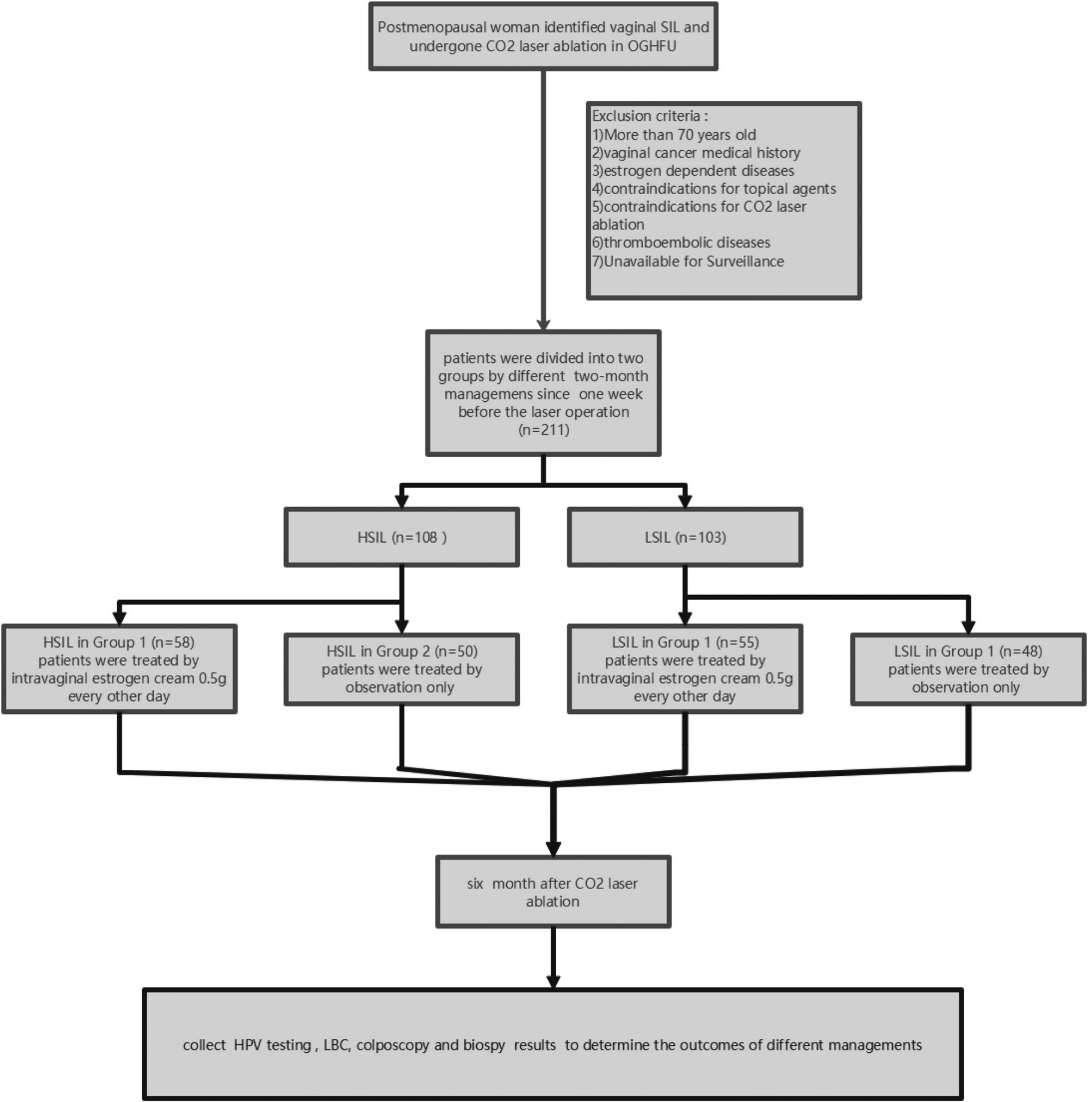
Study flow chart. Abbreviation: OGHFU, Obstetrics and Gynecology Hospital of Fudan University; HPV, human papillomavirus; LBC, liquid-based cytology; COVID-19, corona virus disease 2019.


The clinical data of 211 postmenopausal patients were reviewed from May 2020 to August 2020 in the Center of Diagnosis and Treatment of Cervical Disease, Obstetrics and Gynecology Hospital of Fudan University. Inclusion criteria are as follows: (1) postmenopausal women, (2) proven vaginal SIL by histopathology and the highest-grade lesion was recorded as the final pathologic diagnosis when a range of severity on biopsy, (3) received CO_2_ laser ablation as the initial treatment modality. Exclusion criteria are as follows: (1) more than 70 years old; (2) suspected of invasive vaginal cancer or with vaginal cancer medical history; (3) with or suspected of estrogen-dependent tumors (endometrial cancer, breast cancer, etc.) as well as endometrial dysplasia; (4) with contraindications for intravaginal estrogen or *Lactobacillus*; (5) with contraindications for CO_2_ laser ablation; (6) with thrombophlebitis or thromboembolic diseases; and (7) unavailable for surveillance. Finally, 211 participants were eligible for analysis (Figure 1).

**Informed consent:** Informed consent has been obtained from all individuals included in this study.
**Ethical approval:** The research related to human use has been complied with all the relevant national regulations, institutional policies and in accordance with the tenets of the Helsinki Declaration, and has been approved by the Ethics Committee of the Obstetrics and Gynecology Hospital of Fudan University.

### 2.2 Agent and groups

During the 2-month management since 1 week before the laser operation, some patients used the vaginal estrogen cream, Conjugated Estrogens Cream, Honglilai^®^ (batch number: H20051718), containing 0.625 mg/g estrogen, which has been approved by the China Food and Drug Administration and was widely demonstrated to be safe and effective in helping the vaginal microenvironment in the short term. According to different management strategies, patients were divided into two groups: Group 1 (intervention group): patients were treated with intravaginal estrogen cream 0.5 g every other day; Group 2 (control group): patients were treated by observation only.

### 2.3 Surveillance

Surveillance, including human papillomavirus (HPV) testing, cytology results, and colposcopy histopathology, was finished 6 months after the CO_2_ laser ablation operation to assess the efficacy of the topical agent. Generally, surveillance should be done in the same medical center. However, a few patients were followed up nearby due to the COVID-19 pandemic. Their data were collected by telephone interviews.

### 2.4 Cytology

Liquid-based cytology (LBC) samples collected by experienced gynecologists from the cervical transformation area and the posterior vaginal fornix or vaginal roof were obtained with the SurePath platform (BD Diagnostics) rotating 5–10 turns in the same direction to ensure sufficient number of cells. The sample preparation process in the pathology laboratory was strictly monitored. Cytology results were interpreted and reported by two senior pathologists referring to the 2014 Bethesda System. Slides were reviewed again, and a consensus diagnosis was obtained when differences arose.

### 2.5 HPV testing

HPV testing in the pathology laboratory was performed using a fluorescence-based multiplex real-time HPV DNA genotyping kit (Bioperfectus, Jiangsu, China), which could detect the 14 high-risk HPV genotypes (HPV-16, -18, -31, -33, -35, -39, -45, -51, -52, -56, -58, 59, -66, and -68) with the same sample cells collection method as cytology.

### 2.6 Colposcopy and biopsy

Patients received a colposcopy evaluation 6 months after finishing the initial CO_2_ laser ablation operation. In our research, the Leisegang d-10625 colposcope was used by a specialist to inspect the vulva, vagina, and cervix (if present) according to the manufacturer’s instructions. Tissues showing a white appearance with acetic acid, abnormal blood vessels, non-iodine-staining parts, and suspicious lesions were biopsied and then sent for histopathological examination in the pathology laboratory. Two senior gynecologic pathologists made the diagnosis according to the fourth edition of the World Health Organization (WHO) Classification of Female Genital Tumors.

### 2.7 Observation of clinical therapeutic effects

By comprehensively measuring the laboratory results and biopsy diagnoses before and after treatment, we developed a form of vaginal SIL outcome. Four types of classifications were used: cure, remission, persistence/recurrence, and progression. Among them, the former two were considered effective outcomes, and the remaining were ineffective; more details are provided in Table 1. The primary outcome was to evaluate the rate of effectiveness. The secondary outcome measured the HPV clearance rate.

**Table 1:** Vaginal SIL outcome evaluation form

Pathology of colposcopy biopsy		Cytology	HPV infection	Cervix excision	Outcome
Lesion positive	Downgrade*	/	/	/	Remission
	Same-grade*	Downgrade*	/	/	Persistence/recurrence
	Same-grade*	Upgrade*	/	/	Progression
	Upgrade*	/	/	/	Progression
Lesion negative		≤ASCUS	N	/	Cure
		≤ASCUS	Y	/	Cure (persistent HPV infection)
		>ASCUS	/	N	Cure (abnormal cells from cervix)
		Same-grade*	/	Y	Persistence/recurrence
		Upgrade*	/	Y	Progression

Y: yes. N: no. /: not be considered. *: compared with the results before CO_2_ laser ablation. ASCUS: atypical squamous cell of undetermined significance.

### 2.8 Statistical analysis

Statistical analysis was performed using SPSS (version 26.0; SPSS, Inc., Chicago, USA), indicated as mean (SD) or number (%) of cases, if necessary. Pearson’s chi-square test and Fisher’s exact test were adopted to compare the rates among the groups when suitable. Kendall’s tau method was used to reveal the relationship between lesion severity and efficacy. All tests used were two-tailed. Statistical significance was set at *P* < 0.05.

## 3 Results

Medical records were evaluated for demographic and histopathological characteristics of patients, as presented in Table 2. A total of 211 postmenopausal patients were included in the analysis, 103 vaginal LSIL patients (55 in Group 1, 48 in Group 2), and 108 HSIL patients (58 in Group 1, 50 in Group 2). Four of the HSIL patients (two in Group 1, two in Group 2) developed invasive vaginal cancer; the malignancy rate was 1.9% (4/211). The age range of the study population was 48–69 years, with the median age of diagnosis of 58 years (58 ± 5.4). No difference was found in the following aspects: age at the first sexual intercourse, number of sexual partners, the mode of delivery, and smoking. In our study, patients with cervical lesion history were more likely to suffer from vaginal HSIL. Compared with LSIL patients, the number of patients with cervical HSIL history in vaginal HSIL was more than twice (*P* = 0.001), and the number of patients with cervical cancer history was nearly ten times (*P* < 0.001). Previous studies [16,17] indicated the association between cervical lesions and vaginal intraepithelial neoplasia. Furthermore, we found that cervical HSIL and cervical cancer could increase the severity of vaginal SIL, with correlation coefficient (CC) values of 0.21 (*P* = 0.01) and 0.224 (*P* < 0.001), respectively. In addition, H-R HPV-positive patients accounted for nearly 90% in the HSIL group while accounting for slightly more than 70% (*P* < 0.001). The multiple infection rates in different pathological groups were not significantly different.

**Table 2:** Clinical characteristics of the postmenopausal patients with vaginal SIL

Clinical characteristics	LSIL (103)	HSIL (108)	*P*-Value
Age (mean + SD, years)	58.3 ± 5.4	53.9 ± 9.5	<0.001
Age of first sexual intercourse (mean + SD, years)	23 ± 2.5	23 ± 2.5	0.548
Amount of sexual partners (mean + SD)	1.4 ± 0.6	1.5 ± 0.7	0.793
Smoking (*n*, %)			0.648
Yes	1 (0.01%)	3 (2.8%)	
No	102 (99.9%)	105 (97.2%)	
History of delivery (*n*, %)			0.433
Natural delivery	90 (87.4%)	98 (90.7%)	
Cesarean delivery	13 (12.6%)	10 (9.3%)	
History of hysterectomy (*n*, %)			0.002
Positive	32 (31.1%)	56 (51.9%)	
Negative	71 (68.9%)	52 (48.1%)	
History cervical HSIL (*n*, %)			0.001
Positive	15 (14.6%)	35 (32.4%)	
Negative	88 (85.4%)	73 (67.6%)	
History cervical cancer (*n*, %)			<0.001
Positive	2 (1.9%)	19 (17.6%)	
Negative	101 (98.1%)	89 (82.4%)	
H-R HPV (*n*, %)			<0.001
Positive	73 (70.9%)	100 (92.6%)	
Negative	30 (29.1%)	8 (7.4%)	
H-R HPV positive (*n*, %)			0.135
Single infection	40 (54.8%)	66 (66.3%)	
Multiple infection	33 (45.2%)	34 (33.7%)	

H-R: high risk. LSIL: low-grade squamous intraepithelial lesion. HSIL: high-grade squamous intraepithelial lesion.

Table 3 shows the efficacy evaluation of the two adjuvant treatments after laser ablation. Overall, cure was the primary outcome in different pathological groups regardless of intravaginal medication, which fluctuated from 45% to 55%. Among LSIL patients, the effective rate was 49.1% in the intervention group and 54.2% in the control group (*P* = 0.607); among HSIL patients, the effective rate was 72.4% in the intervention group and 78.0% in the control group (*P* = 0.504). The aforementioned results might suggest that: in different pathological subgroups, estrogen cream could not improve the curative effect. Among patients who received vaginal medication, the response rate was 72.4% (42/58) for HSIL patients and 49.1% (27/55) for LSIL patients (*P* = 0.011); in patients treated by observation, the response rate was 78.0% (39/50) for HSIL patients and 54.2% (26/48) for LSIL patients (*P* = 0.013). The evidence above might further indicate that the response rate for HSIL patients would always be better than that of LSIL patients, which is about 1.5 times that of LSIL patients.

**Table 3:** Outcomes of patients with different vaginal SIL after postoperative management

Groups (*n*)	Treatment outcomes (*n*, %)				Effect (*n*, %)	
	Cure	Regression	Persistence*	Progression	Effective^a^	Ineffective^b^
**LSIL**						
Group 1 (55)	27 (49.1)	0 (0.0)	16 (29.1)	12 (21.8)	27 (49.1)	28 (50.9)
Group 2 (48)	26 (54.2)	0 (0.0)	17 (35.4)	5 (10.4)	26 (54.2)	22 (45.8)
Total (103)	53 (51.0)	0 (0.0)	33 (33.0)	17 (16.0)	53 (51.0)	50 (49.0)
**HSIL**						
Group 1 (58)	29 (50.0)	13 (22.4)	12 (20.7)	4 (6.9)	42 (72.4)	16 (27.6)
Group 2 (50)	22 (44.0)	17 (34.0)	7 (14.0)	4 (8.0)	39 (78.0)	11 (22.0)
Total (108)	51 (47.2)	30 (27.6)	19 (17.6)	8 (7.6)	81 (74.8)	27 (25.2)

Group 1: intravaginal estrogen cream 0.5 g every other day; Group 2: no topical agent.

Persistence*: including persistence and recurrence; effective^a^: including cure and regression.

Ineffective^b^: including persistence, recurrence and progression.

*p*_effect_ = 0.986 when “Group 1 of LSIL” versus “Group 2 of LSIL.”

*p*_effect_ = 0.504 when “Group 1 of HSIL” versus “Group 2 of HSIL.”

*p*_effect_ = **0.030** when “Group 1 of LSIL” versus “Group 1 of HSIL.”

*p*_effect_ = **0.013** when “Group 2 of LSIL” versus “Group 2 of HSIL.”

HPV clearance outcomes of the two groups are given in Table 4. Among all patients included in the analysis, the HPV clearance rate of the estrogen cream users was slightly higher (37.0%, 34/92) than that of the observation group ones (33.3%, 27/81). However, the difference was not statistically significant (*P* = 0.619). By ranking the HPV clearance rate, we found that the order of HPV clearance ability from high to low was Group 2 of LSIL (42.9%, 15/35), Group 1 of HSIL (40.7%, 22/54), Group 1 of LSIL (31.6%, 12/38), and Group 2 of HSIL (21.6%, 12/46). We also stratified analysis of the HPV clearance ability in the four subgroups, but all comparisons did not reveal statistical differences in disease severity and medication.

**Table 4:** HPV status of patients with different vaginal SIL after postoperative management

Group (*n*)	Turned negative (*n*, %)		Persistently positive (*n*, %)		Turn negative rate
	Single	Multiple	Single	Multiple	
**LSIL**					
Group 1 (38)	9 (75.0)	3 (25.0)	11 (42.3)	15 (57.7）	31.2% (12/38)
Group 2 (35)	10 (66.7)	5 (23.3)	10 (50.0)	10 (50.0)	42.9% (15/35)
Total (73)	19 (70.3)	8 (29.7)	21 (45.6)	25 (54.4)	36.99% (27/73)
**HSIL**					
Group 1 (54)	15 (68.2)	7 (31.8)	24 (75.0)	8 (25.0)	40.7% (22/54)
Group 2 (46)	11 (91.7)	1 (8.3)	17 (50.0)	17 (50.0)	26.1% (12/46)
Total (100)	26 (76.5)	8 (23.5)	41 (62.1)	25 (37.9)	34% (34/100)

Group 1: intravaginal estrogen cream 0.5 g every other day; Group 2: no topical agent.

*P*_turn nagative rate_ = 0.178 when “Group 1 of LSIL” versus “Group 2 of LSIL.”

*P*_turn nagative rate_ = 0.123 when “Group 1 of HSIL” versus “Group 2 of HSIL.”

*P*_turn nagative rate_ = 0.199 when “Group 1 of LSIL” versus “Group 1 of HSIL.”

*P*_turn nagative rate_ = 0.113 when “Group 2 of LSIL” versus “Group 2 of HSIL.”

## 4 Discussion

The clinical characteristics of vaginal HSIL make it easier to be identified [18], which may lead to a better therapeutic effect. High-grade lesions show dense acetowhite epithelium, coarse mosaic, coarse punctation, sharp borders, inner border signs, and ridge signs [19]. All of these features help pinpoint the surgical site and enhance laser efficacy. Consistent with that, the effective rate of HSIL patients was about 1.5 times that of LSIL patients in our study. Since the severity of vaginal lesions was the only significant independent factor of persistence/recurrence after one episode of laser [20], we found a phenomenon that the operators are more inclined to enlarge the ablation extent and depth for patients with vaginal HSIL. According to the above, the importance to a CO_2_ laser ablation provider’s ability to evaluate the entire vagina of women with vaginal lesions should be attached.

The decision of CO_2_ laser ablation treatment for vaginal LSIL should be considered. Mengyin Ao et al. [6] retrospectively assessed the largest sample size investigation containing 646 vaginal LSILs with a median follow-up of 14 months (6–60 months). Among the vaginal LSIL patients, none developed invasive vaginal cancer, and the progression rate to vaginal HSIL was 18 (2.8%). Of the 201 LSIL patients treated only by observation, 178 (88.6%) turned to normalization. Since vaginal LSIL generally has a good prognosis [21], it has been suggested that LSIL should be managed conservatively [6,22]. However, in our study population, LSIL patients still had a high progression rate of 49% (50/103). Previous studies have suggested that postmenopausal status, H-R HPV infection, and cervical lesions/cancer history are risk factors for progression [6,9,14,23]. Thus, we recommend active treatments for LSIL women with high risk factors, and more emphasis should be paid to surveillance.

H-R HPV infection is closely associated with high-grade vaginal lesions. H-R HPV was identified in a large proportion of invasive vaginal cancers (70–78%) and almost all vaginal HSILs (92–98%) [14]. In the vaginal HSIL women we reported, the H-R HPV infection rate was as high as 92.6% (100/108). It is well known that postmenopausal women feature estrogen deprivation and *Lactobacillus* absence in the vaginal ecosystem. Estrogen can help vaginal epithelium proliferation and intracellular glycogen production, increasing *Lactobacillus* population [24,25]. With the increase in the *Lactobacillus* population, HPV clearance rate can be significantly improved [26]. We believe that satisfactory management should focus on two aspects: lesion tissue ablation and HPV infection clearance. The latter may be reached through additional supplementation of vaginal probiotics.

**Figure 2:**
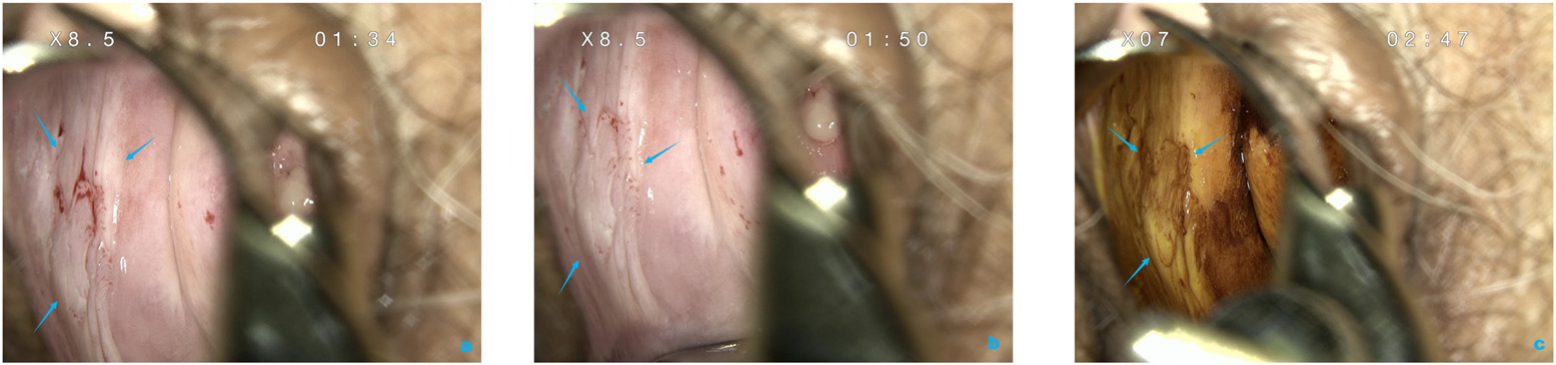
The colposcopy images of a 62-year-old patient who was biopsy proved vaginal LSIL and did not use intravaginal estrogen cream. (a) Vagina without the use of estrogen; (b) acetic acid stained (middle and lower region of the right wall); and (c) iodine-stained.

**Figure 3:**
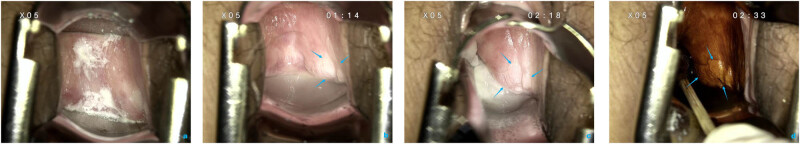
The colposcopy images of a 67-year-old patient after hysterectomy who was biopsy proved vaginal LSIL after two-weeks intravaginal estrogen cream. (a) Vagina after estrogen used; (b) acetic acid stained (upper region of the left wall); (c) enlarged; and (d) iodine-stained.

In postmenopausal patients, although short-term use of intravaginal estrogen cream does not improve the efficacy of the laser, it can increase satisfaction with lesion identification and accelerate the healing after ablation. The lack of estrogen during menopause may negatively impact the vaginal mucosa, resulting in the reduction of elastin and collagen in vaginal tissue, thinning of the vaginal epithelium, and decrease of glycogen in the epithelium [10,27]. All of those changes negatively affect acetowhite epithelium recognition and degree of staining with iodine, especially in vaginal LSIL patients complicated with atrophic vaginitis (Figure 2). In addition, the ablation site often takes longer to heal. During the healing period, some patients may have local adhesion and stenosis, which may bring difficulties in satisfying exposure of the vagina during follow-up examinations. Yet, as shown in Figure 3, we discovered that topical estrogen cream considerably improves such circumstances. In fact, intravaginal estrogen given before colposcopy can improve the elasticity of mucosal folds [13] and present with more reliable colposcopy results [28]. Additionally, postoperative local estrogen management (which began on the day of operation) also resulted in a marked proliferation of the vaginal epithelial layer and active epithelial barrier function [29]. Until now, available evidence presents a complex relationship between estrogen and HPV, with both carcinogenic and anticancer effects on tumor development [30,31,32], while neutral results are presented for estrogen effects in our study. Given the above advantages, we recommend short-term topical estrogen management as a pre-colposcopy preparation and a restorative medication along with CO_2_ laser ablation.

To our best knowledge, this is the largest retrospective study focusing on the potential benefits of using topical estrogen agents along with CO_2_ laser ablation among postmenopausal vaginal SIL patients. Our study is limited. Our sample size is not large enough; further sample collections and elongated follow-up periods may be essential for more accurate analysis. Nevertheless, our study provided a comprehensive analysis, which could add a meaningful insight into vaginal SIL management.

## 5 Conclusions

The treatment of vaginal HSIL in postmenopausal patients with CO_2_ ablation is effective. Short-term intravaginal estrogen combined with CO_2_ laser ablation does not significantly improve the treatment efficacy. Applying estrogen cream locally helps lesion identification under colposcopy and promotes precise treatment.
